# The Spectrum of Central Nervous System Tumors at a Tertiary Care Center Primarily Serving a Rural Population

**DOI:** 10.7759/cureus.57335

**Published:** 2024-03-31

**Authors:** Geeta Maurya, Sanjay K Kannaujia, Rashmi Rashmi, Sanjeev K Singh, Anita Omhare, Roopak Aggarwal

**Affiliations:** 1 Pathology, Uttar Pradesh University of Medical Sciences, Etawah, IND; 2 Pathology, Government Medical College, Kannauj, Kannauj, IND

**Keywords:** who grade, meningioma, astrocytic tumour, neuroepithelial tumour, cns tumours

## Abstract

Background

Central nervous system (CNS) tumors cause significant mortality and morbidity in all age groups. There was no data about the histological spectrum of all CNS tumors in the tertiary care center serving primarily the rural population of Uttar Pradesh.

Aims and objectives

The present study aimed to describe the histopathological spectrum of all CNS tumors reported in a rural tertiary care center at Saifai, Uttar Pradesh. It also aimed to provide an overview of the descriptive epidemiology of CNS tumors.

Material and methods

This was a retrospective, cross-sectional study. The study duration was three years. A total of 115 cases of CNS tumors were studied during that period. Cases were classified according to their histological types, and results were analyzed.

Results

The most common histological group was neuroepithelial tumors, with 53 cases (46.08%). This group had 36 cases of astrocytic tumors (31.3%), three cases of oligodendroglial tumors (2.6%), five cases of oligoastrocytic tumors (4.34%), five cases of ependymal tumors (4.34%), and four cases of embryonal tumors (3.47%). The second most common tumor was meningeal tumors, with 32 cases (27.82%). The male/female ratio (M/F) ratio was 0.7. Females were found to be more affected by almost all histologic categories. Most meningiomas (89.6%) were of World Health Organization (WHO) grade I (26 cases out of 29). Astrocytic tumors showed WHO grade I, II, III, and IV tumors in two cases (5.5%), twelve cases (33.3%), four cases (11.1%), and eighteen cases (50%), respectively. In the younger age group (0-20 years), ependymoma and medulloblastoma were most common, followed by pilocytic astrocytoma and schwannoma.

Conclusion

In this region, neuroepithelial tumors were seen more commonly than meningioma. Females were found to be more affected by CNS tumors. This study has provided relevant data, which can be used for research and better patient management. Further studies with the incorporation of advanced radiological investigation and immunohistochemistry have been recommended.

## Introduction

In India, central nervous system (CNS) tumors account for nearly 2% of all malignancies, and their incidence ranges from 5-10 per 100,000 population [1,2]. CNS tumors are the second most common malignancy after hematopoietic neoplasm during childhood [3]. They are the most common solid malignancy in the pediatric age group [4].

CNS tumors are rare, but they cause significant mortality and morbidity in all age groups. Despite extensive research, no specific risk factors have been identified in the majority of cases [5]. Known risk factors, e.g., heritable genetic syndromes and exposure to ionizing radiation, are seen only in <10% of cases [6].

Many brain tumors are histologically graded from one (I) to four (IV) according to the World Health Organization's (WHO) Classification. However, all brain tumors do not have a classification that runs from grade I to IV (for example, meningiomas are graded from I to III, and a few CNS tumors like hemangiomas are not graded). Histological grading is used for predicting the biological behavior of a tumor. WHO-grade I tumors generally show low proliferative potential, and there is the possibility of a cure with surgical excision only [7]. WHO-grade II tumors also show low levels of proliferative activity, but they are infiltrative in nature. WHO-grade III tumors show histological evidence of malignancy (nuclear atypia, mitosis). WHO-grade IV tumors show malignant features and aggressive behavior, and they are mitotically active, necrosis-prone, and associated with rapid disease evolution [7].

Glioblastoma multiforme (GBM) is a WHO-grade IV tumor with an invasive nature. Despite treatment, less than half of patients survive for more than a year [8].

However, with advances in surgical techniques, radiology, newer chemotherapy, and radiotherapy, treatment outcomes are being improved day by day [9].

To date, there is no data available for CNS tumors in the rural population in this region. This study will give insights into the histopathological spectrum of CNS tumors. This data will be useful for better patient care as well as for further research.

## Materials and methods

This study was conducted in the Department of Pathology, Uttar Pradesh University of Medical Sciences (UPUMS), Saifai, Uttar Pradesh, India. This was a retrospective, cross-sectional, hospital-based descriptive study.

The study duration was three years (from January 2017 to December 2019). The place of study was the Department of Pathology at UPUMS, Saifai. After obtaining approval from the ethical committee of the institution and after applying inclusion and exclusion criteria, a total of 115 biopsies of CNS tumors were retrieved from available histopathology records. The study included all the cases of CNS tumors (cases of brain and spinal cord tumors) that were presented in the Department of Neurosurgery. These surgical biopsies were received in the Department of Pathology and reported during the study period. All the non-neoplastic lesions (inflammatory and cystic lesions) were excluded. All inadequate/insufficient biopsies were also excluded. Case details, along with clinical and radiological records, were noted.

Gross and microscopic examinations were evaluated by the authors. Formalin-fixed paraffin-embedded tissue blocks/hematoxylin and eosine (H&E)-stained slides were retrieved. The H&E-stained slides were reviewed by the authors. After confirming the histopathological diagnosis, all the cases were classified and graded according to WHO Classification 2016. Thus, the histopathological spectrum of CNS tumors could be seen. Generally, many CNS tumors are histologically graded from one (I) to four (IV) according to their behavior. Histological grading is used for predicting the biological behavior of a tumor. Data was analyzed for histopathological spectrum and frequencies in different age groups and genders.

## Results

In this study, we retrieved 115 cases of CNS tumors over three years. Of these, 113 cases (98.26%) were primary tumors, and only two cases (1.74%) were found to be metastatic (secondary tumors) (Table 1).

**Table 1:** Primary and secondary tumors CNS: central nervous system

Sl No	CNS tumors	Frequency
1	Primary tumor	113
2	Secondary tumor	2
Total cases		115

The most common histological types were neuroepithelial tumors (53 cases, 46.08%). Out of 53 neuroepithelial tumor cases, 36 were astrocytic tumors. In astrocytic tumors, nearly half the tumors (17 out of 36 cases) were diagnosed as GBM (WHO grade IV). The second most common CNS tumor category was meningeal tumors (32 cases, 27.82%), followed by tumors of the cranial and paraspinal nerves (16 cases, 13.91%). Meningothelial meningioma (nine cases) was the most common meningioma found in this study (Table 2).

**Table 2:** Histological types of CNS tumors CNS: central nervous system

Sl. No.	WHO Classification categories (number of cases/percentage)		Histologic types	WHO grade	Number of cases	Percentage (%)
1	Tumors of meninges (32 cases/27.82%)	Meningiomas (29 cases/25.21%)	Meningothelial meningioma	I	9	7.82%
			Transitional (mixed) meningioma	I	2	1.74%
			Psammomatous meningioma	I	3	2.60%
			Fibroblastic meningioma	I	5	4.34%
			Angiomatous meningioma	I	6	5.21%
			Microcystic meningioma	I	1	0.86%
			Atypical meningioma	II	2	1.74%
			Rhabdoid meningioma	III	1	0.86%
		Meningeal mesenchymal tumors (3 cases/2.60%)	Hemangioma	NA	1	0.86%
			Hemangioblastoma	I	2	1.74%
2	Neuroepithelial tumors (53 cases/46.08%)	Astrocytic tumors (36 cases/31.30%)	Pilocytic astrocytoma	I	2	1.74%
			Gemistocytic astrocytoma	II	2	1.74%
			Fibrillary astrocytoma	II	8	6.95%
			Diffuse astrocytoma	II	2	1.74%
			Anaplastic astrocytoma	III	4	3.47%
			Glioblastoma	IV	17	14.78%
			Gliosarcoma	IV	1	0.86%
		Oligodendroglial tumors (3 cases/2.60%)	Oligodendroglioma	II	2	1.74%
			Anaplastic oligodendroglioma	III	1	0.86%
		Oligoastrocytic tumors (5 cases/4.34%)	Oligoastrocytoma	II	4	3.47%
			Anaplastic oligoastrocytoma	III	1	0.86%
		Ependymal tumors (5 cases/4.34%)	Ependymoma	II	5	4.34%
		Embryonal tumors (4 cases/3.47%)	Medulloblastoma	IV	4	3.47%
3	Tumors of cranial and paraspinal nerves (16 cases/13.91%)		Schwannoma	I	12	10.43%
			Neurofibroma	I	4	3.47%
4	Hematopoietic neoplasm (3 cases/2.60%)		Lymphoma		1	0.86%
			Plasmacytoma		2	1.74%
5	Germ cell tumors (2 cases/1.73%)		Germinoma		1	0.86%
			Choriocarcinoma		1	0.86%
6	Tumors of the sellar region (7 cases/6.08%)		Craniopharyngioma	I	2	1.74%
			Pituitary adenoma	I	5	4.34%
7	Metastatic tumors (2 cases/1.73%)		Metastatic tumors		2	1.74%
Total					115	100%

In total, 107 cases from this study were graded according to the 2016 WHO Classification. Of these, 53 cases (49.53%) were found to be of grade I. While 25 cases (23.36%), seven cases (6.54%), and 22 cases (20.56%) were diagnosed as grade II, grade III, and grade IV, respectively.

**Figure 1:**
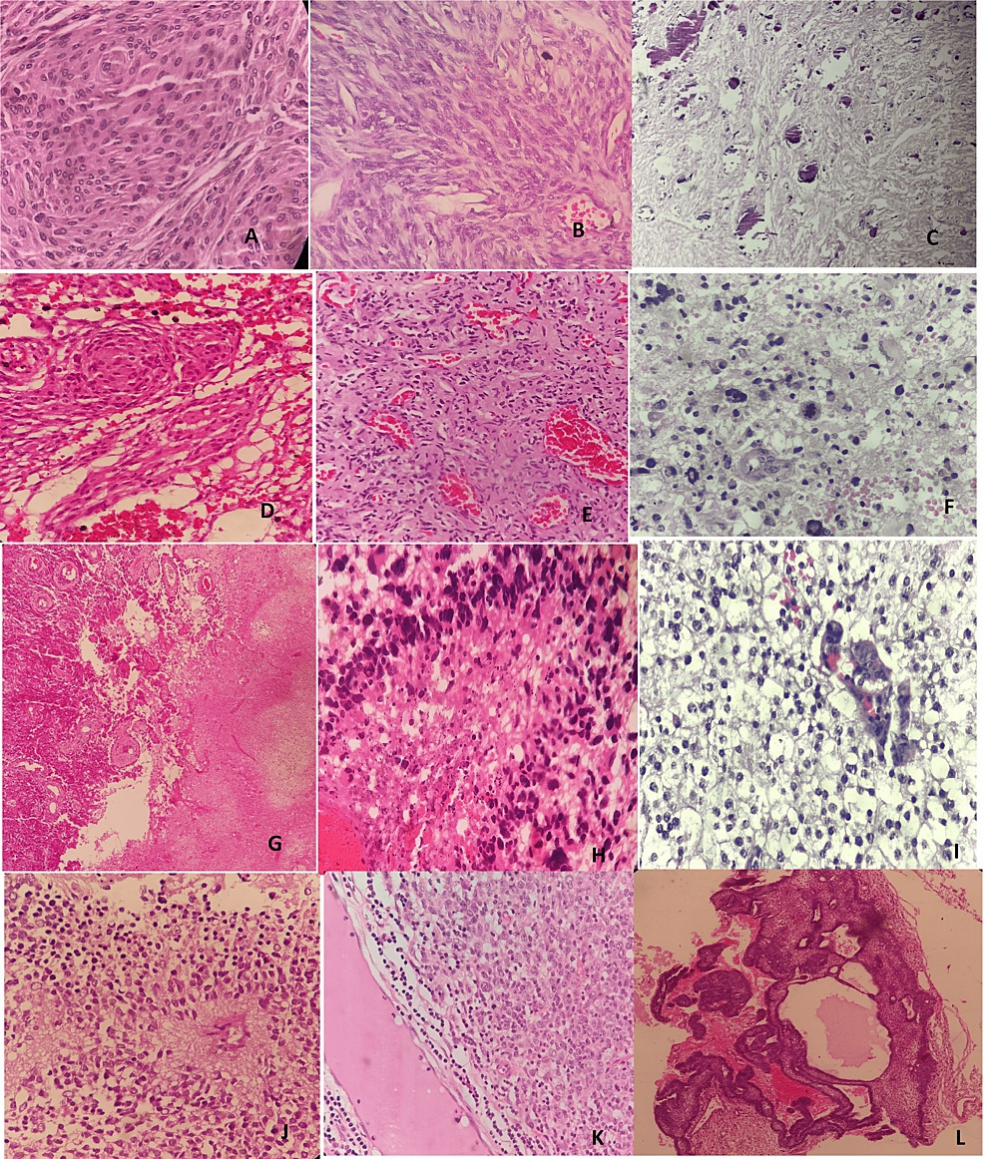
Photomicrographs showing morphological features of common CNS tumors in our study A: Meningothelial meningioma (grade I), lobulated architecture, syncytial cells [H&E, 400x]; B: Fibroblastic meningioma (grade I), spindle cells, bundles of collagen [H&E, 400x]; C: Psammomatous meningioma (grade I), numerous psammoma bodies [H&E, 100x]; D: Mixed meningioma (grade I), mixed meningothelial and fibroblastic type features [H&E, 100x]; E: Angiomatous meningioma (grade I), majority of the section shows vascular component [H&E, 100x]; F: Anaplastic astrocytoma (grade III), distinct nuclear atypia and mitotic activity [H&E, 400x]; G: Glioblastoma (grade IV) showing necrosis [H&E, 40x]; H: Glioblastoma (grade IV), pseudopalisading necrosis [H&E, 400x]; I: Oligodendroglioma (grade III), perinuclear clearing [H&E, 400x]; J: Ependymoma (grade II), perivascular pseudorosettes [H&E, 400x]; K: Lymphoma, diffuse infiltrate of intermediate sized lymphoid cells with scanty cytoplasm [H&E, 400x]; L: Craniopharyngioma, a squamous epithelium with peripheral nuclear palisading [H&E, 100x] CNS: central nervous system; H&E: hematoxylin and eosine

In total, 26 cases of meningioma were found at WHO grade I. Two cases of atypical meningioma (grade II) and one case of rhabdoid meningioma (grade III) were also diagnosed. Astrocytic tumors showed WHO grade I, II, III, and IV tumors in two cases (5.5%), 12 cases (33.3%), four cases (11.1%), and 18 cases (50%), respectively. In grade IV tumors, the most common tumors were glioblastoma with 17 cases, followed by medulloblastoma with four cases, and gliosarcoma with one case (Table 2). The morphological features of common CNS tumors in our study are described in Figure 1. 

In this study, the M/F ratio was 0.7, i.e., overall, females were more affected with 68 cases (59.13%), while the number of male patients was 47 cases (40.87%). In meningioma, females were three times more affected (22 out of 29 cases, 75.86%) than males. Tumors of the cranial and spinal nerves also showed female gender predilection in 11 out of 16 cases (68.75%). Cases of neuroepithelial tumors were distributed equally among both genders (Table 3). 

**Table 3:** Gender-wise distribution

Sl. No.	Histologic categories		Male patient	Female patient	Total
1	Tumors of the meninges				
	(a)	Meningiomas	7	22	29
	(b)	Meningeal mesenchymal tumors	1	2	3
2	Neuroepithelial tumors				
	(a)	Astrocytic tumors	19	17	36
	(b)	Oligodendroglial tumors	2	1	3
	(c)	Oligoastrocytic tumors	2	3	5
	(d)	Ependymal tumors	0	5	5
	(e)	Embryonal tumors	3	1	4
3	Tumors of cranial nerves and paraspinal nerves		5	11	16
4	Hematopoietic neoplasm		2	1	3
5	Germ cell tumors		0	2	2
6	Tumors of sellar region		4	3	7
7	Metastatic tumors		2	0	2
Total			47 (40.87%)	68 (59.13%)	115 (100%)

We found the greatest number of CNS tumor cases in the age group 41-50 years (total 31 cases, 26.95%), followed by the age group 21-30 years (26 cases, 22.34%), and 31-40 years (20 cases, 17.39%). Astrocytic tumors were found to be most prevalent in the age group 41-50 years (Table 4). 

**Table 4:** Age-wise distribution

Sl. No.	Histologic categories	Age group 0-10 years	Age group 11-20 years	Age group 21-30 years	Age group 31-40 years	Age group 41-50 years	Age group 51-60 years	Age group >60 years	Number of cases
1	Meningiomas	0	0	8	8	8	3	2	29
2	Meningeal mesenchymal tumors	0	1	1	0	1	0	0	03
3	Astrocytic tumors	1	2	8	8	12	3	2	36
4	Oligodendroglial tumors	0	0	2	1	1	3	1	8
5	Ependymal tumors	2	2	0	0	1	0	0	5
6	Embryonal tumors	2	2	0	0	0	0	0	4
7	Tumors of cranial nerves and spinal nerves	0	3	3	2	4	3	1	16
8	Hematopoietic neoplasm	0	0	0	0	2	1	0	3
9	Germ cell tumors	1	0	1	0	0	0	0	2
10	Tumors of sellar region	0	2	3	0	2	0	0	7
11	Metastatic tumors	0	0	0	1	0	0	1	2
	Total cases (%)	6 (5.21%)	12 (10.43%)	26 (22.60%)	20 (17.39%)	31 (26.95%)	13 (11.30%)	7 (6.08%)	115 (100%)

In our study, a total of 18 patients with CNS tumors were found in patients <20 years of age, of whom eight were males and 10 were females (Table 5).

**Table 5:** Distribution of CNS tumors among children and younger age group (0-20 years) SOL: space-occupying lesion; CNS: central nervous system

Sl. No.	Age	Sex	Histologic subtype	WHO grade	Location
1	6 years	Male	Pilocytic astrocytoma	I	Posterior fossa tumor
2	18 years	Male	Pilocytic astrocytoma	I	Posterior fossa tumor
3	17 years	Male	Glioblastoma	IV	Frontal lobe space occupying lesion (SOL)
4	5 years	Female	Ependymoma	II	Posterior fossa tumor
5	6 years	Female	Ependymoma	II	Posterior fossa tumor (4^th^ ventricular mass)
6	17 years	Female	Ependymoma	II	Posterior fossa tumor
7	20 years	Female	Ependymoma	II	Posterior fossa tumor
8	6 years	Male	Medulloblastoma	IV	Posterior fossa SOL
9	8 years	Female	Medulloblastoma	IV	4^th^ ventricular mass
10	12 years	Male	Medulloblastoma	IV	Posterior fossa SOL
11	15 years	Male	Medulloblastoma	IV	Posterior fossa SOL
12	18 years	Female	Schwannoma	I	Extradural paravertebral
13	14 years	Male	Schwannoma	I	Extradural paravertebral
14	18 years	Female	Neurofibroma	I	Extradural paravertebral
15	9 years	Female	Germinoma		Suprasellar extending to 3^rd^ ventricle
16	11 years	Male	Craniopharyngioma	I	Suprasellar SOL
17	19 years	Female	Pituitary adenoma	I	Suprasellar SOL
18	15 years	Female	Hemangioma		Impinge secondarily upon CNS

The most common CNS tumors encountered in this age group (<20 years) were ependymoma and medulloblastoma, with four cases each. The most common location of tumors was the posterior fossa.

## Discussion

CNS tumors are rare, but they are the second most common malignancy in children after leukemia. In this study, we reviewed 115 cases of CNS tumors retrospectively to categorize them according to WHO histological grades.

Many similar studies showed meningioma as the most common CNS tumor. Bhattacharya et al. (2022) [10] and Sen et al. (2022) [11] found meningeal tumors to be 42.86% and 48% of all CNS tumors, respectively. In this study, the most common CNS tumors were neuroepithelial tumors (46.08%), followed by meningeal tumors (27.82%).

In a statistical report by Ostrom et al. (2022), the most common malignant brain tumor was glioblastoma (14.2% of all CNS tumors) [12]. Similarly, glioblastoma was the most common malignant tumor in our study, with 17 cases (14.78% of all CNS tumors and 47.2% of astrocytic tumors), and the median age was 42 years. Thambi et al. (2017) stated that the median age for glioblastoma was 55.5 years [13]. Interestingly, in this study, we have reported glioblastoma in a 17-year-old male.

Many studies showed male patients' predominance in CNS tumors (Table 6), while in this study overall, females were more affected than males in almost all histologic categories of CNS tumors, especially in meningioma, as 75.86% of cases were female. Similarly, meningiomas were seen more commonly in females by Sen et al. (2022) [11] and Lee et al. (2010) [14]. In this study, the M/F ratio was 0.7. Thambi et al. (2017) also found a lower M/F ratio (0.9) [13]. In a study by Jaiswal et al. (2016) [15], the most frequent tumor histology among children was astrocytomas (25.1%), followed by embryonal (20.6%), and ependymal tumors (14.8%). Chen et al. (2013) [16] observed astrocytomas as the most common tumor (29.2%) in this age group (0-19 years), followed by medulloblastoma (13.1%). Our results were comparable, as the most common tumors were ependymal tumors (22.2%) and medulloblastoma (22.2%), i.e., four cases out of 18 each in the younger age group (0-20 years).

**Table 6:** Comparison of common variables in cited studies [10,11,13,17]

Studies	Bhattacharya et al.	Sen et al.	Thambi et al.	Ghanghoria et al.	This study
Number of cases	42	42	510	65	115
M/F ratio	1.2/1	1.5/1	0.9/1	1.16/1	0.7/1
Most common histological types of tumors	Meningeal - 42.86% Neuroepithelial - 38.09%	Meningeal - 48% Astrocytic - 38%	Meningioma - 34.7% Astrocytic - 25.1%	Meningioma - 41.54% Astrocytic - 24.61%	Meningioma - 27.82% Astrocytic - 31.30%
Most common age group	Sixth decade	21-40 years	40-60 years	30-50 years	41-50

Astrocytic tumors and cranial and paraspinal nerve tumors were three cases each (16.6%). We compared our results with those of other similar studies, and the comparison results have been tabulated (Table 6).

However, our study has limitations because it was conducted in one center only. Biopsies of patients undergoing surgery in the hospital are included in the study, so this may not be representative of the entire population/rural population of this region.

## Conclusions

Through this study, we came to know about the histopathological spectrum of CNS tumors in the rural region of Uttar Pradesh. We observed the frequencies of different histological types with their WHO grading, sex, and age distribution. This study provided relevant data that can be used for academic information and research. Further studies with the incorporation of advanced radiological investigation and immunohistochemistry are required for comprehensive research and better patient management.
